# Supplementary material for: On variability in local field potentials
Source: bioRxiv. 2025 Apr 2:2025.03.27.645661. Preprint. [Version 2] doi: 10.1101/2025.03.27.645661 (PMC11974936; doi:10.1101/2025.03.27.645661)
Supplement: Supplement 1 [file media-1.pdf]

Supplementary information

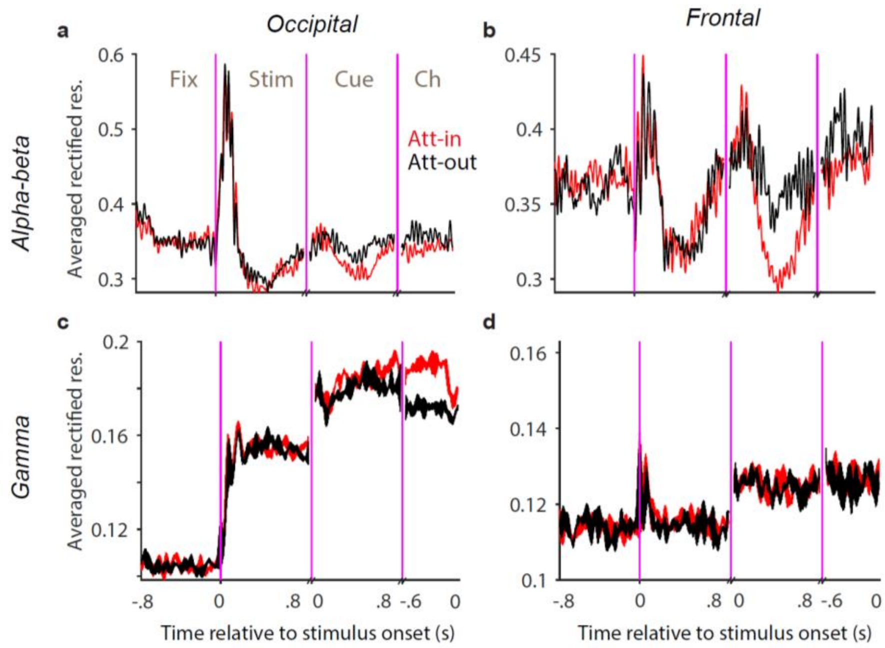

Figure S1. The rectified LFP averaged over trials reflects ATV of LFP.

a, b) The trial-averaged rectified LFP, after filtering in the alpha-beta band (8–18 Hz) for attend-IN (red) and attend-OUT (black) conditions, shown for the occipital (**a**) and frontal (**b**) cluster.
c, d) Same as (**a, b**), but for data filtered in the gamma band (70–80 Hz).

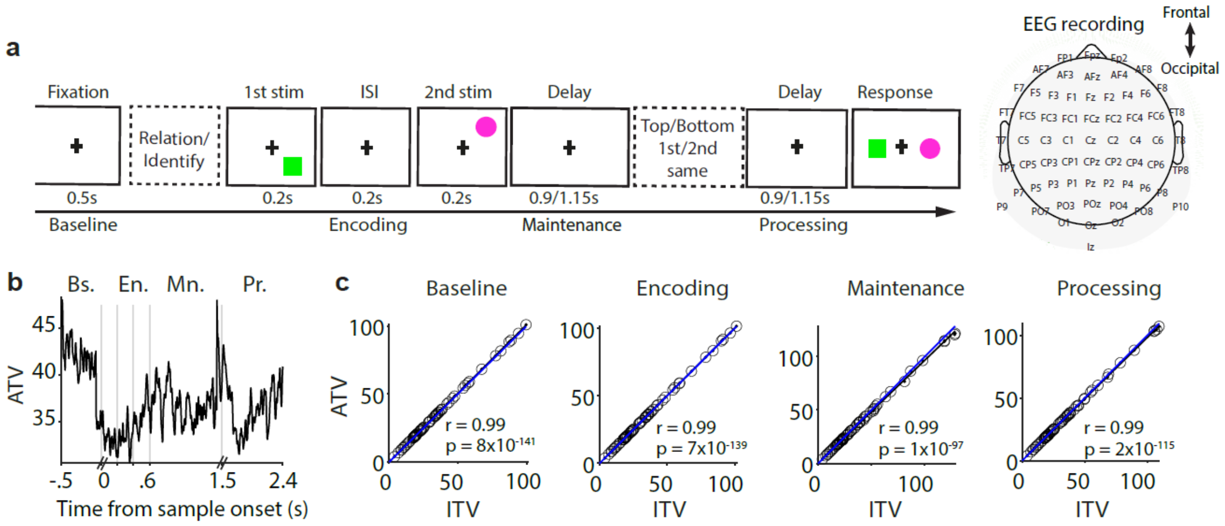

Figure S2. ATV correlates with ITV for EEG data.

a) Schematic of the behavioral working memory (WM) task consisting of four task periods: baseline, encoding, maintenance, and active processing. Subjects maintained the spatial and temporal features of stimuli while performing the WM task. Data were recorded from 64 EEG electrodes.

b) Time-course of ATV during the four task periods for one example subject averaged across 64 recording channels. *Bs*: baseline, *En*: encoding, *Mn*: maintenance, and *Pr*: active processing.

c) Correlation between ATV and ITV during the four task periods. Each dot represents data from one recording channel.
